# Development and Application of a Modified Biochar-Calcium Alginate Composite (MB-CA) for In Situ Remediation of Cadmium-Contaminated Soil

**DOI:** 10.3390/gels11050375

**Published:** 2025-05-20

**Authors:** Sijia Sun, Yuying Wang, Yanru Zhang, Lina Wu, Xinyi Wang, Guoyu Wang, Weitao Sun, Dasong Lin, Yajun Wang

**Affiliations:** 1 College of Resources and Environment Sciences, Northeast Agricultural University, Harbin 150030, China; 15149938192@163.com; 2 Agro-Environmental Protection Institute, Ministry of Agriculture and Rural Affairs, Tianjin 300191, China; 3 College of Agriculture and Forestry Technology, Weifang Vocational College, Weifang 262737, China; wyy0624@126.com; 4 Resource Environment, Tianjin Agricultural University, Tianjin 300384, China

**Keywords:** modified biochar, calcium alginate, Cd pollution, in situ passivation

## Abstract

Agricultural monitoring reveals cadmium (Cd) as the most prevalent heavy metal pollutant in Chinese agricultural soils, with 7.0% of sampled sites exceeding the national soil environmental quality standard (GB 15618-2018), creating substantial risks for crop safety. In situ remediation is a cost-effective method that can modify the speciation and migration properties of Cd in soil. The previous stage of research studies conducted basic characterization of materials and predicted their adsorption capacity in solution environments. This study focuses on the application effects in soil environment. We cross-linked modified biochar and calcium alginate hydrogels to fabricate a composite material (MB-CA) and determined its excellent adsorption performance for cadmium. This study is a continuation of our previous work, focusing on determining the thermodynamic model of adsorption materials, the applicable environment of composite materials, the influence on soil microorganisms, and its effect on the reduction in Cd content in agricultural products. The research found that the adsorption of Cd^2+^ by MB-CA conforms to the Freundlich isotherm model. MB-CA has the ability to regulate pH, achieving outstanding adsorption capacity at pH 4–6. The effect of MB-CA on lettuce is verified through pot experiment and field experiment. The Cd^2+^ content in plants decreased by 63.11% and 76.92%, respectively. Additionally, MB-CA did not negatively impact microbial abundance. This study further discussed the performance and application effect of MB-CA, providing new solutions for soil remediation.

## 1. Introduction

Soil serves as the fundamental substrate for sustainable agricultural development. However, China’s arable lands have experienced severe cadmium (Cd) contamination over the past four decades, driven by rapid industrialization, intensive agricultural practices, and insufficient environmental regulations [1]. Among the pollutants, cadmium (Cd) is the most prevalent heavy metal in Chinese agricultural soils, with 7.0% of sampled sites exceeding the national soil environmental quality standard (GB 15618-2018), significantly higher than that of other inorganic pollutants [2]. Soil contamination with heavy metal Cd is highly hazardous due to its high toxicity and difficulty to remove [3]. Cd can disrupt the normal absorption of essential nutrients by plants, inhibit photosynthesis, and affect their growth and metabolism. Additionally, Cd is toxic to human kidneys, bones, and the respiratory system, and is classified as a Group 1 carcinogen [4]. Therefore, maintaining Cd-free soils is essential to ensure ecological safety and human health [5].

To address soil Cd pollution, researchers worldwide have conducted extensive studies and proposed various remediation technologies, including physical, chemical, and biological approaches, as well as integrated remediation strategies [6,7]. Among these, in situ immobilization using soil amendments has emerged as a cost-effective method [8]. The common heavy metal passivation materials include biochar, lime, shell powder, etc. They can alter the chemical speciation and mobility of heavy metals through mechanisms such as ion exchange, surface complexation, and microbial redox reactions.

Hydrogels are three-dimensional polymeric networks formed through chemical or physical cross-linking mechanisms, where individual or multiple homopolymers/copolymers undergo molecular interconnection to establish a stable architecture [9]. Due to their wide availability, low-cost, and strong adsorption capacity for metal ions, hydrogels have been widely applied in the absorption of heavy metal ions [10]. Various functional groups in the hydrogel network, such as hydroxyl (-OH), amino (-NH_2_), carboxyl (-COOH), and amide (-CONH_2_) groups enable complexation reactions with Cd^2+^ ions, effectively removing heavy metal Cd from soil [11,12,13]. He et al. developed a starch-based composite hydrogel using calcium–magnesium minerals, demonstrating significant Cd adsorption capacity with a maximum adsorption capacity of 591.36 mg/g [14]. Sun et al. synthesized a novel polyacrylamide/acrylic acid/vinyl imidazole bromide (PAM/AA/[Vim]Br^2−^) hydrogel as an adsorbent for removing Ni^2+^, Cu^2+^, Zn^2+^ and Cr^3+^ from water. The results show that the hydrogel exhibits a multilayer of physical adsorption mechanism and a monolayer of chemical adsorption mechanism, achieving heavy metal removal rates exceeding 80–90% [15]. Sun et al. studied alginate composite gels and found that they primarily adsorb metal ions through electrostatic interactions, ion exchange, chelation, complexation, and reduction reactions [11]. Alginate gels exhibit great potential in environmental remediation due to their high efficiency, biodegradability, and cost-effectiveness [16].

Biochar is a traditional soil conditioner, and its passivation effect on heavy metals can be greatly improved by modification [17]. The incorporation of organic/inorganic components into alginate-based matrices has been widely adopted for engineering hydrogel systems with tunable functional attributes, effectively optimizing key parameters including swelling kinetics, stress–strain behavior, and adsorption capacity [18]. Embedding modified biochar into an alginate matrix can significantly improve the adsorption properties of alginate-based composite materials [19,20]. Cataldo et al. used activated carbon–calcium alginate composite materials to adsorb lead, showing the maximum adsorption capacity with 15.7 mg/g, which was a notable improvement compared to 10.7 mg/g for activated carbon alone [21].

In the previous work, we cross-linked modified biochar and calcium alginate hydrogels to fabricate a composite material (MB-CA) and determined its excellent adsorption performance for cadmium [10]. This study serves as a supplement and continuation of prior research, with a specific emphasis on delineating the thermodynamic model of adsorption materials, identifying the suitable environmental conditions for composite materials, assessing the impacts on soil microbial communities, and evaluating their effectiveness in reducing Cd accumulation levels in agricultural products. This study deeply analyzed the properties and application effect of MB-CA and offers new solutions for the field of soil remediation.

## 2. Results and Discussion

### 2.1. The Interaction Between pH and MB-CA

**Figure 1:**
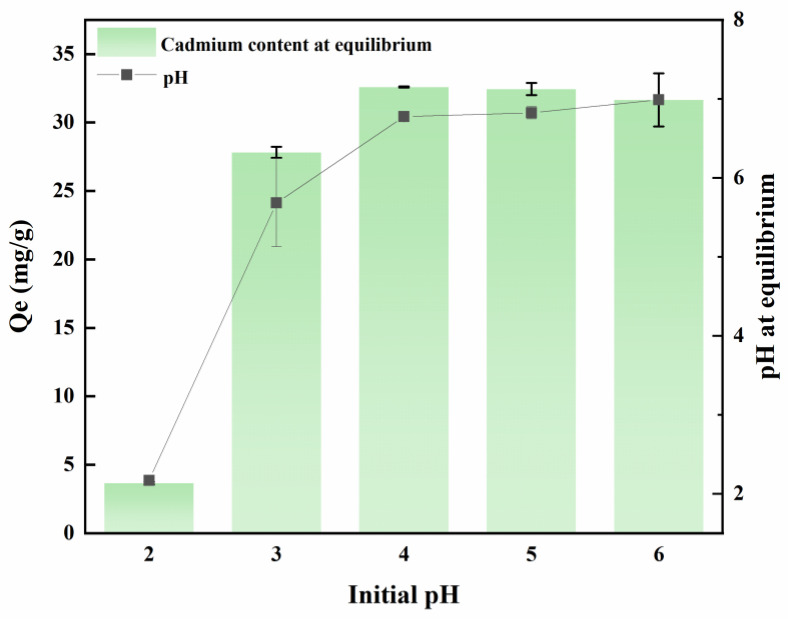
The interaction between pH and MB-CA.

As depicted in Figure 1, the pH level exerts a substantial influence on the adsorption of MB-CA. Commencing from an initial pH range of 2 to 6, the adsorption capacity progressively escalates, ultimately achieving a steady state at 30 mg/g. In an environment with a pH of 2, the solution is abundant in H^+^ ions. These ions readily adsorb onto the surface of MB-CA, occupying a substantial proportion of the available adsorption sites. Simultaneously, Cd^2+^ exists in the form of cations and competes with H^+^ ions on the surface of the adsorption material, leading to a decrease in cadmium adsorption efficiency at a pH of 2. As the pH value gradually increases, the concentration of H^+^ ions decreases accordingly, reducing competitive interactions and allowing the composite material MB-CA to reach its maximum adsorption capacity. The maximum adsorption capacity at pH 2 is 3.65 mg/g, whereas at pH 4, it significantly increases to 32.59 mg/g, demonstrating a remarkable enhancement in adsorption capacity.

Additionally, the experiment found that MB-CA has the ability to regulate the pH of the solution, bringing the equilibrium pH closer to neutral. This phenomenon can potentially be ascribed to the fact that calcium alginate is a salt formed from a strong base and a weak acid, coupled with the inherently alkaline physicochemical properties of biochar. Whether the effect of Cd adsorption and raising pH of MB-CA are also excellent in practical applications needs to be verified by potted and field experiments.

In pot and field experiments, comparative trials were conducted among the composite material (MB-CA), biochar (MB) and the control group. As shown in Table 1, showed that both MB and MB-CA can increase soil pH value, but the MB-CA performed better, raising the soil pH from 6.27 to 6.76 and 6.92, respectively. This result confirms that the composite material MB-CA has a significant effect on improving the pH value of soil, which provides a new way to reduce the bioavailability of Cd in soil. It indicated that this composite material demonstrates significant potential for application in the remediation of acidic soils and the control of heavy metal contamination.

### 2.2. Isothermal Adsorption Experiment of MB-CA

In this research, the Langmuir and Freundlich isothermal adsorption models were used to fit the experimental data, as shown in Equations (1) and (2), respectively:

qc_l_ = q_m_k_L_Ce/(1 + k_1_Ce) (1)

qc_f_ = K_F_Ce^n^ (2)

where, qc_l_ is the Langmuir adsorption equation, qc_f_ is the Freundlich adsorption equation, k_L_ is a constant related to adsorption energy, and q_m_ is the maximum adsorption capacity under Langmuir monolayer adsorption conditions (mg/kg); K_F_ is the Freundlich adsorption characteristic constant representing adsorption capacity, and n is the Freundlich adsorption characteristic constant representing adsorption strength.

**Figure 2:**
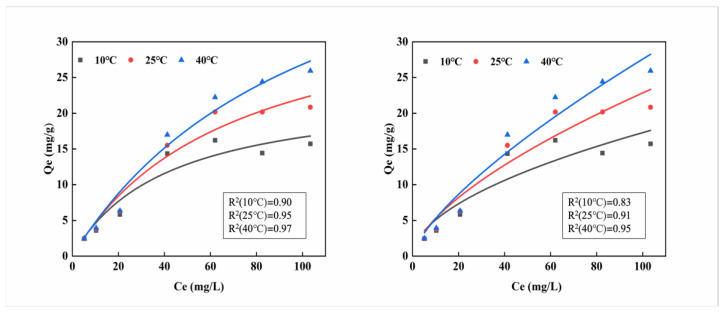
Isothermal adsorption fitting curves of MB-CA at three temperatures (10 °C, 25 °C, 40 °C) ((**a**): Langmuire, (**b**): Freundlich).

The adsorption line isotherm illustrates the interaction between adsorption equilibrium and pollutant concentration, from which the maximum adsorption capacity can be analyzed. As shown in Figure 2, the maximum adsorption capacity gradually increases with the increase of cadmium concentration. When the solution reaches its highest concentration, the curve continues to rise, indicating that the maximum adsorption capacity has not been reached. In the Langmuir curve, the maximum adsorption capacity at 40 °C is the highest at 25.94 mg/g. The favorability of the Freundlich isotherm is usually indicated by the magnitude of the exponent, which also reaches its maximum value of 25.94 mg·g^−1^ at 40 °C. The adsorption capacity gradually increases with temperature, suggesting that the calcium alginate material has better adsorption effectiveness around 40 °C.

This study used the Langmuir and Freundlich models to analyze different adsorption isotherms. Based on an examination of the fitting coefficients (R^2^) of the two models, it is evident that the R^2^ values for the Freundlich model surpass those of the Langmuir model across all three temperatures. This observation suggests that the Freundlich model provides a more accurate description of the adsorption process of Cd^2+^ on MB-CA, rendering it the more suitable of the two models for this specific application. Calculations using the Freundlich model show that the maximum adsorption capacity of MB-CA is more consistent with the experimental maximum adsorption capacity, suggesting that the adsorption of Cd^2+^ on MB-CA is a multilayer adsorption process [22].

### 2.3. The Impact of Different Treatments on the Forms of Cd in Soil

The different forms of heavy metal Cd represent its various modes of existence in the soil and reflect its potential environmental behavior and biological effects. Exchangeable cadmium, being highly mobile, is susceptible to various environmental influences, thereby facilitating its migration and transformation within ecosystems. This form of cadmium often exhibits high bioavailability, making it easily absorbable and prone to accumulation in plant tissues. In the pot experiments, exchangeable Cd accounted for 24.91% of the total Cd in the tested soil, while the proportions of carbonate-bound and iron–manganese oxide-bound Cd were relatively less. The application of modified biochar did not significantly affect the different forms of soil Cd. Compared to the control group, the application of the composite material MB-CA reduced the proportion of exchangeable Cd from 24.91% to 17.65%. The proportion of residual Cd increased from 58.13% to 67.4%, while the carbonate-bound and iron–manganese oxide-bound forms showed no significant changes.

**Figure 3:**
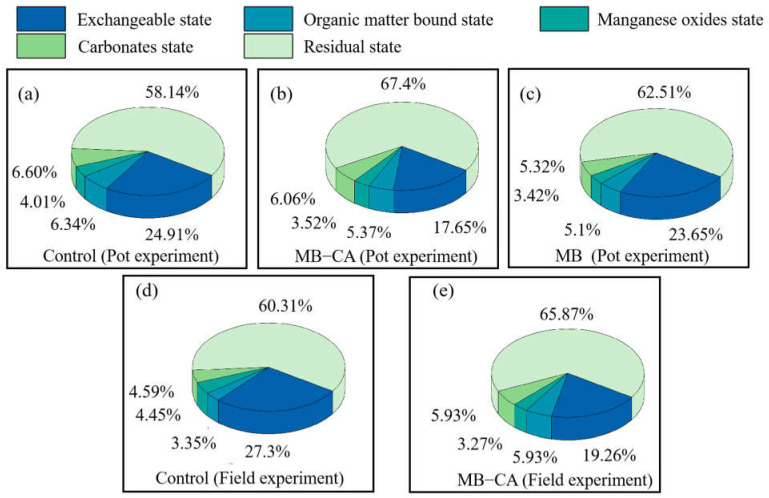
Effects of each treatment on soil Cd morphology in pot and field experiments.((**a**): Control (Pot experiment), (**b**): MB-CA (Pot experiment), (**c**): MB (Pot experiment), (**d**): Control (Field experiment), (**e**): MB-CA (Field experiment)).

In the field experiments, the different forms of Cd underwent transformations in the soil. During the 40-day observation period, the content of exchangeable Cd showed a continuous decline, while the content of residual Cd gradually increased. Notably, the application of MB-CA had a particularly significant impact on exchangeable Cd. As shown in Figure 3d,e, compared to the control group, the content of exchangeable Cd in the MB-CA treatment group decreased from 27.3% to 19.26%. The increase in the proportion of residual Cd is consistent with the results from the soil experiments, indicating that the addition of the composite material made Cd^2+^ in the soil more stable.

**Figure 4:**
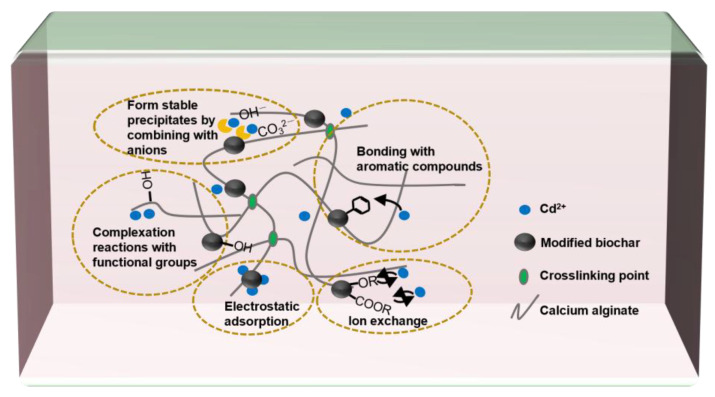
Adsorption mechanism of Cd^2+^ by MB-CA.

As shown in Figure 4, the mechanism in which the MB-CA reduces the Cd content in the soil mainly involves the combination of anions such as OH^−^ and ${\mathrm{CO}}_{3}^{2-}$ with Cd^2+^, or the complexation reactions between numerous functional groups in the MB-CA with Cd^2+^. According to research by Wang et al. [10], calcium alginate is rich in functional groups such as -COOH and -OH, which can rapidly coordinate with cadmium ions, thereby reducing the activity of heavy metal cadmium. In addition, the adsorption mechanism of Cd^2+^ also includes bonding with aromatic compounds, ion exchange with itself metal ions, and physical adsorption [23].

### 2.4. The Effects of Different Treatments on the Content of Cadmium in Soil and Plants

**Figure 5:**
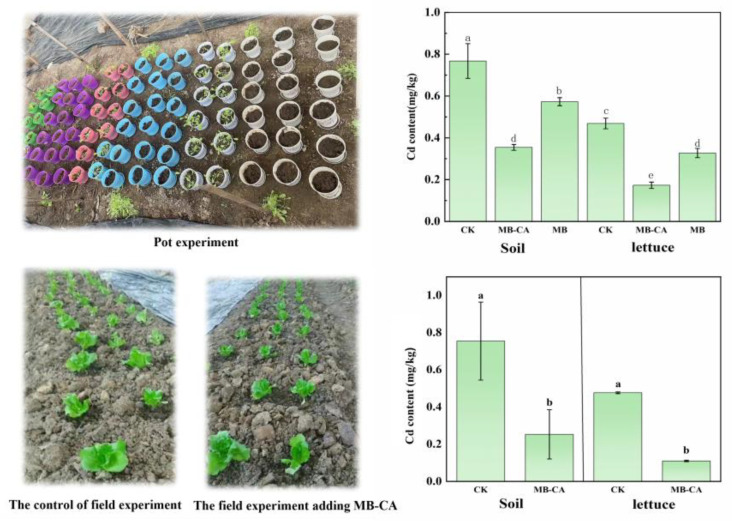
The effects of each treatment on cadmium content in soil and lettuce in pot and field experiments. (Different lowercase letters indicate significant differences, *p* < 0.05).

In the pot and field experiments, the Cd content in the tested soil was approximately 0.75 mg/kg, which is higher than the pollution risk screening value for agricultural land soils specified in GB15618-2018 (0.3 mg/kg, 6.5 < pH ≤ 7.5). As shown in Figure 5, compared to the control group, the application of the MB-CA reduced the soil Cd content from 0.76 mg/kg to 0.35 mg/kg and 0.25 mg/kg, respectively, as the soil was tested after the composite material was removed. This indicates that the composite material adsorbed a large amount of Cd^2+^ onto its surface and interior, effectively reducing the Cd content in the soil and lowering the risk of Cd transfer from the soil to agricultural products. This is primarily due to the fact that the MB-CA combines the dual advantages of biochar and calcium alginate, possessing both the adsorption properties of biochar and the ion exchange and chelation capabilities of calcium alginate, thereby achieving efficient adsorption of Cd^2+^.

The Cd content in lettuce plants showed significant differences under various treatments. As illustrated in Figure 5, the modified biochar (MB) treatment also demonstrated a certain passivation effect, but the composite material MB-CA had the most significant impact on reducing the Cd content in lettuce. Specifically, in field and pot experiments, MB-CA treatment reduced the cadmium content in lettuce by 63.11% and 76.92%, respectively, compared to the control group, while the MB treatment reduced it by 33.10%. The composite material MB-CA completed the adsorption and accumulation process of the heavy metal Cd in the soil, effectively reducing the Cd content in the soil and decreasing the bioavailability of soil Cd. The addition of the material significantly reduced the Cd content in the edible parts of the plants.

### 2.5. Effect of MB-CA on Soil Microorganisms

**Figure 6:**
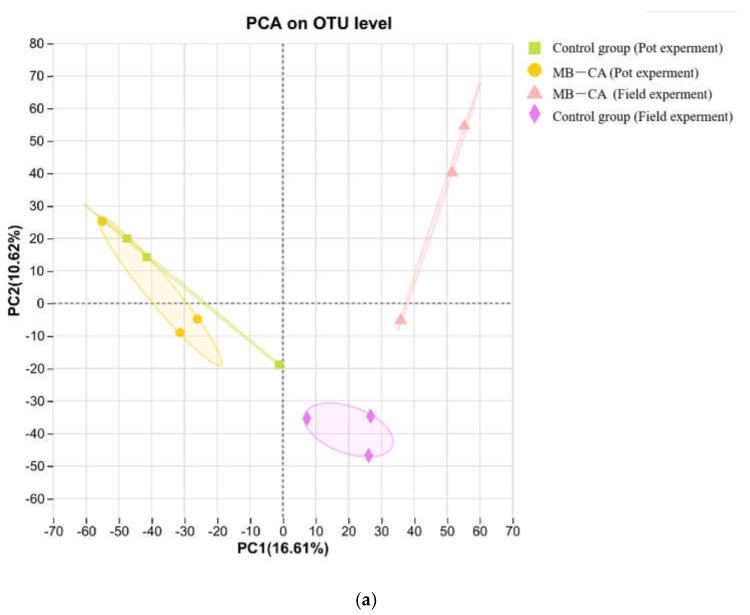

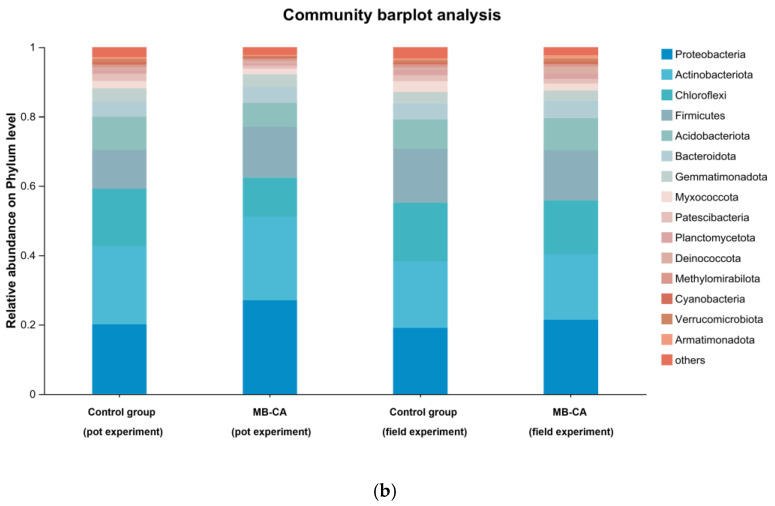
Microbial PCA analysis (**a**) and community bar chart (**b**) in pot and field experiments.

As shown in Table 2, the microbial coverage rate was around 98%, indicating that the depth of gene sequencing could represent changes in the microbial community. According to the PCA results (Figure 6), the original field soil plotted in the fourth quadrant differed from the soil samples with added MB-CA. This is because calcium alginate has water retention and film-forming properties, which can improve soil aggregates and promote microbial activity. Meanwhile, biochar can stimulate microbial growth in the short term. The effect of this experiment was poorer in pot trials, with the original soils from pots and fields located in the first and second quadrants, respectively. The reason may be the lower microbial activity in the pots. The Chao1 index of the soil with added composite material MB-CA increased by 4% and decreased by 3%, respectively, indicating that this material does not negatively affect the relative abundance of microorganisms in the soil.

The main microbial genera in the soil are Proteobacteria, Actinobacteriota, Chloroflexi, and Firmicutes. The addition of material MB-CA had a certain promoting effect on the main bacterial genera in the soil. For example, the relative abundance of Proteobacteria increased by 5.9% and 1.3%, respectively.

## 3. Conclusions

In this study, a composite material MB-CA was prepared by cross-linking and intercalating modified biochar with calcium alginate. This material demonstrated superior adsorption and passivation capabilities for cadmium in arable land, providing a sustainable method to remove heavy metals. pH is an important factor affecting the adsorption efficiency, with the maximum adsorption capacity increasing with rising pH. In addition, it was demonstrated that the composite material MB-CA has a potential for regulating soil pH. Isothermal adsorption experiments showed that the Freundlich model provided a better fit than the Langmuir model, making it more suitable for describing the Cd adsorption process, indicating that the adsorption of Cd^2+^ by MB-CA is a multi-layer heterogeneous adsorption.

The composite material MB-CA effectively reduced the content of heavy metal cadmium in the soil and decreased the available forms of cadmium. By applying MB-CA to the soil, it was found that the material could improve the soil pH, with experimental results consistent with those in solution, gradually trending towards neutrality. In the Tessier experiment, the content of exchangeable Cd decreased from 27.3% to 19.26% after applying MB-CA, and the proportion of residual forms increased. Pot experiments showed that both MB-CA and MB could reduce cadmium content in soil and plants, with the cadmium content in lettuce decreasing by 63.11% after applying MB-CA and by 33.10% with MB treatment.

In microbial experiments, the PCA results revealed that the application of the composite material MB-CA in pot experiments did not significantly alter the microbial samples. However, in field experiments, there were differences observed. Microbial diversity indices indicated that the Chao1 index of the soil increased by 4% and decreased by 3% after applying the material in pots and fields, respectively, showing that MB-CA does not inhibit the abundance of soil microorganisms obviously. Community bar charts showed that the abundance of major microbial populations in the soil increased after applying the material MB-CA. This research provides valuable empirical support for the application of hydrogel in agricultural production. It should not be ignored that the calcium alginate component of this composite material is prone to being decomposed by microorganisms in the soil. How to extend its inhibition time for heavy metals is the key task for this research in the future.

## 4. Materials and Methods

### 4.1. Preparation of Materials

(1) Preparation of Modified Biochar (MB): Acid modification is a widely recognized and effective chemical modification approach [24,25,26,27]. Pine wood chips were crushed and mixed with a 40% V/V phosphoric acid solution at a solid-to-liquid ratio of 2:5. The mixture was thoroughly stirred and impregnated at 40 °C for 12 h to ensure complete penetration of the phosphoric acid into the wood chips. Subsequently, the mixture was dried for later use. The dried mixture was then subjected to slow pyrolysis in a tube furnace under a nitrogen atmosphere (final pyrolysis temperature of 750 °C, held for 1 h). After the furnace cooled completely, the modified biochar was collected and sieved through a 60-mesh screen. Biochar of uniform particle size was washed with deionized water and dried for further use.

(2) Preparation of Modified Biochar-Calcium Alginate Hydrogel Composite (MB-CA): A total of 6.0 g of modified biochar was weighed and mixed with 200 mL of a 2% calcium alginate solution. The mixture was subjected to ultrasonic stirring for 60 min to ensure uniform dispersion of the modified biochar within the calcium alginate solution. Ultrasonic stirring effectively promotes the dispersion and mixing of components in the solution, ensuring even distribution of the modified biochar. Subsequently, the mixed solution was slowly dripped into a calcium chloride solution. The cross-linking effect of calcium chloride caused the calcium alginate and modified biochar to co-solidify into spherical hydrogel beads. After 60 min of cross-linking, the spherical hydrogel beads gradually settled at the bottom of the beaker, forming the composite material MB-CA. The composite material was then washed with deionized water to remove potential impurities and unreacted components. Finally, the washed composite material was cooled and dried.

### 4.2. Pot Experiment

Based on previous studies, adsorption kinetics and soil cultivation experiments have demonstrated that calcium alginate (MB-CA) exhibits excellent remediation effects. To further validate its practical application performance, this study conducted pot and field experiments. The pot and field experiments described in this study were carried out in Xiangtan Comprehensive Experimental Station, Institute of Environmental Protection Research and Monitoring, Ministry of Agriculture and Rural Affairs. The experimental station is located in Xiangtan City, Hunan Province, at a geographical location of 112°53′ E and 27°52′ N, with an altitude of approximately 40 m. The Cd-contaminated soil used in the experiments was collected from Cd-polluted paddy fields in Xiangtan City, Hunan Province. The relevant physicochemical properties of the soil are presented in Table 3.

The pot experiment included four treatment groups. At the beginning of the experiment, 50 g of Cd-contaminated soil was weighed for each treatment. A composite material, equivalent to 2% of the soil weight, was added to each treatment group. To compare the effectiveness of the composite material, 2 control groups were established: (1) a mixture of Cd-contaminated soil and modified biochar (added 2% of the soil weight); and (2) a blank control group sprayed only with deionized water. Each treatment group was replicated 3 times to ensure the reliability and stability of the results.

Lettuce seeds were sown, with approximately 10 seeds per pot. After the lettuce seedlings developed their first true leaves, thinning was performed, retaining two seedlings with similar growth vigor in each pot to minimize the impact of individual differences on the experimental results. After a period of 40 days, the lettuce was carefully harvested, and various indicators, including fresh weight and Cd content, were meticulously measured and recorded. Additionally, soil samples from each treatment group were collected (using the same method as in the soil incubation experiment). The chemical forms of Cd in the soil were analyzed using the Tessier sequential extraction technique, and the total Cd content in the soil was determined using graphite furnace atomic absorption spectrometry to evaluate the adsorption efficiency of the composite material on Cd^2+^.

### 4.3. Field Experiment

The field experiment included two following treatments: (1) application of modified biochar-calcium alginate hydrogel composite material at a rate of 1 kg/m^2^; and (2) a control group consisting of Cd-contaminated soil sprayed only with deionized water. During the experiment, normal irrigation was maintained. Soil samples were collected at the beginning of the experiment and after 40 days of using the five-point sampling method. The samples were analyzed to evaluate the Cd adsorption efficiency of the composite material and its impact on the chemical forms of Cd in the soil. Additionally, the fresh weight of lettuce and the Cd content in the edible parts were measured.

### 4.4. Soil pH Measurement

Soil pH was measured using the potentiometric method, with water as the extractant and a soil-to-water ratio of 2.5:1. The pH of the sample suspension was determined at 25 °C using a pH meter.

### 4.5. Total Cd Content Analysis

Soil sampling was conducted using the five-point method, sieved through a 100-mesh sieve. The digestion process is as follows: an analytical balance with a precision of 0.0001 g was used to weigh 0.2000 g of soil sample into a Teflon digestion tube. Then, 9 mL of nitric acid-hydrochloric acid-perchloric acid mixture (9:3:2), 3 mL of hydrochloric acid, and 2 mL of hydrofluoric acid were added to the digestion tube, which was placed in a graphite digestion instrument (LabTech ED 54-itouch Digestion System). After digestion, the solution was fixed to volume with deionized water. An atomic absorption spectrometer was employed for determination. Each treatment was sampled three times for quality control.

After the lettuce reached maturity, lettuce samples were collected and initially rinsed with tap water to remove surface soil and impurities. To further ensure the purity of the samples, the lettuce was rinsed with ultrapure water. Finally, 50 g of tender leaves from each plant were selected, homogenized into a slurry, and used for Cd content determination.

Detection method of the total Cd: approximately 0.2500 g of dried plant sample was weighed and placed in a digestion tube. A mixture of 8 mL concentrated nitric acid and 1 mL perchloric acid was added, and the sample was left to stand overnight. The following day, the digestion tube was placed in an electric heating digestion instrument (LabTech, DigiBlock ED54, Beijing, China) for digestion. After digestion, the sample was filtered and analyzed using atomic absorption spectrometry.

### 4.6. Soil Cd Speciation Analysis

The Tessier sequential extraction method [28] was used to analyze the chemical forms of Cd in the soil, including the exchangeable fraction, carbonate-bound fraction, iron–manganese oxide-bound fraction, organic matter-bound fraction, and residual fraction. The specific extraction steps are detailed in Table 4.

### 4.7. MB-CA Isothermal Adsorption Experimental Method

The isothermal adsorption experiment of MB-CA was conducted using a constant-temperature water bath to control the environmental temperature. Cd(NO_3_)_2_ solutions with concentrations of 5, 10, 20, 40, 60, 80, and 100 mg/L were prepared, and their initial pH was adjusted to 5.0 using 0.1 mol/L NaOH and HCl solutions. A volume of 50 mL of each solution was transferred to a beaker, and 60 mg of MB-CA was added to each beaker. The beakers were placed in a constant temperature water bath, and the reaction temperatures were set to 25, 35, 45, and 55 °C. The adsorption time was set to 60 min. After reaching the adsorption time, 10 mL of the solution was sampled three times at each temperature for further analysis [29].

### 4.8. Effect of pH on MB-CA Adsorption Efficiency

In accordance with the methodology outlined by Sun et al., a solution of Cd(NO_3_)_2_ was prepared, maintaining a concentration of 50 mg/L. The initial pH of the solution was adjusted to 2.0, 3.0, 4.0, 5.0, and 6.0 using HCl and NaOH solutions [30]. A volume of 30 mL of each pH-adjusted solution was transferred to centrifuge tubes. Then, 60 mg of dried MB-CA composite material was added to each tube. The tubes were placed in a constant temperature shaker and agitated at 25 ± 1 °C for 24 h. After allowing the reaction system to reach equilibrium for 12 h, approximately 2 cm below the liquid surface was sampled three times using a pipette. After being filtered and diluted, the samples were subjected to atomic absorption spectrometry analysis to ascertain the concentration of Cd^2+^ and to assess the effectiveness of the composite material in removing Cd under varying pH conditions.

### 4.9. Measurement Methods for Soil Microorganisms

The FastPure Soil DNA Isolation Kit (Magnetic bead, MJYH, Shanghai, China) was used for DNA extraction. Total microbial genomic DNA was extracted from the samples using the E.Z.N.A.^®^ Soil DNA Kit (Omega Bio-tek, Norcross, GA, USA). The quality and concentration of the DNA were assessed using 1.0% agarose gel electrophoresis and a NanoDrop 2000 spectrophotometer (Thermo Scientific, New York, NY, USA). Finally, sequencing was performed using the Nextseq 2000 sequencing platform (Illumina, New York, NY, USA).

### 4.10. Reagents

The reagents used in this study are shown in Table 5.

## Figures and Tables

**Table 1:** Effects of each treatment on soil pH levels in potted plants and field experiments.

Test	Treatment		
	CK	MB-CA	MB
Pot experiment	6.27 ± 0.06	6.76 ± 0.07	6.47 ± 0.07
Field experiment	6.51 ± 0.05	6.92 ± 0.05	6.79 ± 0.12

**Table 2:** Microbial Diversity Index in Pot and Field Experiments.

Treatments		Community Diversity		Community Richness		Coverage
		ACE	Chao1	Shannon	Simpson	
Pot experiment	Control group	4150.92 ± 473.57	4040.25 ± 474.00	6.740	0.005	0.981
	MB-CA	4115.93 ± 84.51	3911.92 ± 73.7	6.466	0.006	0.981
Field experiment	Control group	4397.61 ± 294.55	4266.04 ± 284.15	6.751	0.005	0.980
	MB-CA	4219.60 ± 133.98	4100.24 ± 176.15	6.593	0.005	0.980

**Table 3:** Physicochemical properties of the tested soil.

pH	Soil Organic Matter g/kg	CEC mol/kg	Cd Content mg/kg
6.77 ± 0.20	40.6	9.1	0.73

**Table 4:** Tessier detection method.

Heavy Metal Speciation	Solution	Condition
Exchangeable state (T1)	MgCl_2_ (1 mol/L), pH = 7.0	25 °C oscillations 1 h
Organic matter bound state (T2)	NaAc (1 mol/L), pH = 5.0	25 °C oscillations 8 h
Manganese oxides state (T3)	NH_2_OH·HC1(25% HAc, 0.04 mol/L)	96 °C oscillations 4 h
Carbonates state (T4)	30% H_2_O_2_, pH= 2.0 NH_4_Ac (20% HNO_3_, 3.2 mol/L)	85 °C oscillations 2 h 25 °C oscillations 30 min
Residual state (T5)	HNO_3_-HF	Dissolve until completely dissolved

**Table 5:** Experimental reagents.

Reagent Name	Purity Level	Source
C_6_H_7_NaO_6_ (SA)	Chemical pure	Sinopharm Chemical Reagent Co., Ltd., Shanghai, China
CaCl_2_	Chemical pure	Bodi Chemical Industry Co., Ltd., Tianjin, China
NaOH	Analytial reagent	Sinopharm Chemical Reagent Co., Ltd., Shanghai, China
Cd(NO_3_)_2_·4H_2_O	Analytial reagent	Fuchen Chemical Reagent Co., Ltd., Tianjin, China
NaNO_3_	Analytial reagent	Xinhao Chemical Industry Co., Ltd., Zibo, China
HNO_3_	Analytial reagent	Luxi Chemical Industry Group Co., Ltd., Liaocheng, China
H_3_PO	Analytial reagent	Taixi Chemical Industry Co., Ltd., Jinan, China
HCl	Analytial reagent	Beiyuan Chemical Industry Group Co., Ltd., Yulin, China

## Data Availability

The original contributions presented in this study are included in the article. Further inquiries can be directed to the corresponding authors.

## References

[1] Shi B.L., Zhang X.H., Gou A.P. Research on heavy metal pollution remediation technology in farmland soil. Proceedings of the 2nd International Conference on Geoscience and Environmental Chemistry (ICGEC).

[2] Luo Y., Teng Y. (2020). Research Progresses and Prospects on Soil Pollution and Remediation in China. Acta Pedol. Sin..

[3] Lata S., Kaur H.P., Mishra T. (2019). Cadmium Bioremediation: A Review. Int. J. Pharm. Sci. Res..

[4] Yu X.L., He Y. (2022). Positive Effects and Optimal Ranges of Tea Saponins on Phytoremediation of Cadmium-Contaminated Soil. Sustainability.

[5] Wang L., Cui X., Cheng H., Chen F., Wang J., Zhao X., Lin C., Pu X. (2015). A review of soil cadmium contamination in China including a health risk assessment. Environ. Sci. Pollut. Res..

[6] Su H., Cai Z., Zhou Q.X. Phytoremediation of Cadmium Contaminated Soils: Advances and Researching Prospects. Proceedings of the Chinese Materials Congress (CMC 2012).

[7] Luo N., Zhang X.J., Gong L.F., Yang L.Q., Yao Q., Song J.F. (2025). Review of Remediation Methods for Soil Contaminated with Cadmium. Eurasian Soil Sci..

[8] Lombi E., Hamon R.E., McGrath S.P., McLaughlin M.J. (2003). Lability of Cd, Cu, and Zn in polluted soils treated with lime, beringite, and red mud and identification of a non-labile colloidal fraction of metals using isotopic techniques. Environ. Sci. Technol..

[9] Berradi A., Aziz F., El Achaby M., Ouazzani N., Mandi L. (2023). A Comprehensive Review of Polysaccharide-Based Hydrogels as Promising Biomaterials. Polymers.

[10] Wang S.Y., Wang Y.J., Wang X.Y., Sun S.J., Zhang Y.R., Jiao W.X., Lin D.S. (2024). Study on Adsorption of Cd in Solution and Soil by Modified Biochar-Calcium Alginate Hydrogel. Gels.

[11] Sun R., Gao S., Zhang K., Cheng W.-T., Hu G. (2024). Recent advances in alginate-based composite gel spheres for removal of heavy metals. Int. J. Biol. Macromol..

[12] Pandey A., Bera D., Shukla A., Ray L. (2007). Studies on Cr(VI), Pb(II) and Cu(II) adsorption-desorption using calcium alginate as biopolymer. Chem. Speciat. Bioavailab..

[13] Gao X.P., Guo C., Hao J.J., Zhao Z., Long H.M., Li M.Y. (2020). Adsorption of heavy metal ions by sodium alginate based adsorbent-a review and new perspectives. Int. J. Biol. Macromol..

[14] He C.Q., Mou H.Y., Hou W.J., Chen W.Q., Ao T.Q. (2024). Drought-resistant and water-retaining tobermorite/starch composite hydrogel for the remediation of cadmium-contaminated soil. Int. J. Biol. Macromol..

[15] Sun J., Jin Z.Q., Wang J.Y., Wang H., Zhang Q., Gao H.J., Jin Z.H., Zhang J.L., Wang Z.W. (2023). Application of Ionic Liquid Crosslinked Hydrogel for Removing Heavy Metal Ions from Water: Different Concentration Ranges with Different Adsorption Mechanisms. Polymers.

[16] Gao Q.J., Wang X., Wang H.X., Liang D.X., Zhang J.G., Li J. (2019). Sulfhydryl-modified sodium alginate film for lead-ion adsorption. Mater. Lett..

[17] Wang B., Gao B., Wan Y. (2018). Entrapment of ball-milled biochar in Ca-alginate beads for the removal of aqueous Cd(II). J. Ind. Eng. Chem..

[18] Badsha M.A.H., Khan M., Wu B., Kumar A., Lo I.M.C. (2021). Role of surface functional groups of hydrogels in metal adsorption: From performance to mechanism. J. Hazard. Mater..

[19] Zhang Y.Q., Fan B.G., Jia L., Qiao X.L., Li Z.P. (2022). Study on adsorption mechanism of mercury on Ce-Cu modified iron-based biochar. Chem. Eng. J. Adv..

[20] Sanyang L., Ghani W., Idris A., Bin Ahmad M. Zinc Removal from Wastewater Using Hydrogel Modified Biochar. Proceedings of the 3rd International Conference on Process Engineering and Advanced Materials (ICPEAM).

[21] Cataldo S., Gianguzza A., Milea D., Muratore N., Pettignano A. (2016). Pb(II) adsorption by a novel activated carbon—Alginate composite material. A kinetic and equilibrium study. Int. J. Biol. Macromol..

[22] Hameed B.H., Tan I.A.W., Ahmad A.L. (2008). Adsorption isotherm, kinetic modeling and mechanism of 2,4,6-trichlorophenol on coconut husk-based activated carbon. Chem. Eng. J..

[23] Qin J., Fang Y.T., Ou C.J., Wang J.Y., Huang F., Wen Q., Liao Z.P., Shi J. (2023). Highly efficient Cd^2+^ and Cu^2+^ removal by MgO-modified tobermorite in aqueous solutions. J. Environ. Chem. Eng..

[24] Li F.Y., Xie Y., Wang Y., Fan X.J., Cai Y.B., Mei Y.Y. (2019). Improvement of dyes degradation using hydrofluoric acid modified biochar as persulfate activator. Environ. Pollut. Bioavailab..

[25] Wang J., Shi D.Y., Huang C.Z., Zhai B.Y., Feng S.Y. (2023). Effects of Common Biochar and Acid-Modified Biochar on Growth and Quality of Spinach in Coastal Saline Soils. Plants.

[26] Shao C.H., Fan F., Dai Y.J. (2024). Lead ions removal from water by tartaric acid modified biochar materials: Equilibrium, kinetic studies and mechanism. Desalination Water Treat..

[27] Xu Y.G., Bai T.X., Yan Y.B., Zhao Y.F., Yuan L., Pan P., Jiang Z. (2020). Enhanced removal of hexavalent chromium by different acid-modified biochar derived from corn straw: Behavior and mechanism. Water Sci. Technol..

[28] He Q.S., Ren Y., Mohamed I., Ali M., Hassan W., Zeng F.G. (2013). Assessment of Trace and Heavy Metal Distribution by Four Sequential Extraction Procedures in a Contaminated Soil. Soil Water Res..

[29] Agrafioti E., Kalderis D., Diamadopoulos E. (2014). Arsenic and chromium removal from water using biochars derived from rice husk, organic solid wastes and sewage sludge. J. Environ. Manag..

[30] Sun X.Z., Guo P., Sun Y.Y., Cui Y.Q. (2021). Adsorption of Hexavalent Chromium by Sodium Alginate Fiber Biochar Loaded with Lanthanum. Materials.

